# Changes in breakfast frequency and composition during adolescence: The Adolescent Nutritional Assessment Longitudinal Study, a cohort from Brazil

**DOI:** 10.1371/journal.pone.0200587

**Published:** 2018-07-19

**Authors:** Bruna Kulik Hassan, Diana Barbosa Cunha, Gloria Valeria da Veiga, Rosangela Alves Pereira, Rosely Sichieri

**Affiliations:** 1 Department of Epidemiology, Institute of Social Medicine, State University of Rio de Janeiro (UERJ), Rio de Janeiro, Rio de Janeiro, Brazil; 2 Department of Social and Applied Nutrition, Institute of Nutrition Josué de Castro, Federal University of Rio de Janeiro (UFRJ), Rio de Janeiro, Rio de Janeiro, Brazil; Faculty of Medicine and Health Sciences, SWEDEN

## Abstract

**Objectives:**

To estimate changes over time of breakfast frequency and foods/food groups consumed at breakfast.

**Methods:**

Cohort of 809 students aged 10–16 years old from Rio de Janeiro, Brazil, enrolled in 2010, and followed for three years. Breakfast skippers were those not eating breakfast. Those with breakfast frequency of 4 or less times per week were considered irregular breakfast eaters. Changes over time of breakfast frequency and breakfast foods/food groups were analyzed by generalized estimating equations.

**Results:**

At baseline, overweight/obese girls had higher prevalence of irregular breakfast than those non-overweight/obese (40% vs. 26%; p = 0.005); among boys, there was no significant difference in the prevalence of irregular breakfast according to weight status. After three years, among girls there was an increase in the risk of irregular breakfast consumption (RR = 1.29; 95% CI: 1.08; 1.54) and breakfast skipping (RR = 1.63; 95% CI: 1.12; 2.38). Also, overweight/obese boys (RR = 1.40, 95% CI: 1.03, 1.89) and non-overweight/obese girls (RR = 1.54, 95% CI: 1.17, 2.04) had higher risk of irregular breakfast in three years, compared to baseline. After three years, boys changed the consumption of foods/food groups at breakfast and presented higher risk of decreased intake of fruits (RR = 1.60, 95% CI: 1.20, 2.13), sandwiches and snacks (RR = 1.58, 95% CI: 1.12, 2.22), chips (RR = 1.43, 95% CI: 1.01, 2.13), and ham (RR = 1.52, 95% CI: 1.12, 2.07), and lower risk of cheese intake (RR = 0.73, 95% CI: 0.53, 1.00). Girls had higher risk of decreased intake of fruits (RR = 2.08, 95% CI: 1.47, 2.95), milk (RR = 1.49, 95% CI: 1.07, 2.08), chocolate powder (RR = 1.54, 95% CI: 1.11, 2.14) and ham (RR = 1.65, 95% CI: 1.16, 2.36).

**Conclusions:**

Changes in breakfast patterns are different according to sex. Also, different changes in breakfast frequency according to BMI category were found. Consumption patterns of some foods/food groups have a tendency to become changed from initial to middle adolescence.

## Introduction

The practice of eating breakfast regularly among adolescents range from 51% to 95% worldwide [1–5], whilst 7% to 32% of them skip this meal or consume it irregularly [2,4,6–10]. This wide range in prevalence may be partly explained by how this meal is defined and analyzed. While some studies define breakfast consumption by time [8,11,12] and/or prior to performing other daily activities [2,12], others substitute or add to their definition some specific foods [2,5,9,13], or even a caloric percentage of the total daily energy requirements [12,14].

A frequency of at least five times a week is used to mark a regular frequency of breakfast eating [2,4], though lower [12,15] or higher cutoffs [3] have also been used. Also, cutoff points for skipping breakfast and irregular breakfast vary between studies. For example, Sjöberg et al. (2003) defined irregular breakfast intake as omission of this meal at least once a week [2]. Another study categorized as skippers those eating breakfast less than 3 days a week and “semi-skippers” those with breakfast frequency of 3 to 4 days a week [4]. Timlin et al. (2008) divided adolescent’s breakfast eating in three groups: (1) daily eaters, for those who responded “every day”; (2) irregular eaters, with breakfast frequency between 1 to 6 days per week and; (3) daily skippers, for those who responded “never” [16]. Therefore, despite being a consensus that breakfast is the first meal of the day [12,17], there is no clear and standardized definition or categorization when concerns breakfast frequency.

Breakfast frequency has been related to several health benefits and healthier lifestyle factors. Observational studies suggest frequent breakfast in adolescence to be associated with better academic and cognitive performance [6,18,19], regular family meal frequency [20], lower body fatness, lower fasting glucose levels, higher cardiorespiratory fitness and healthier cardiovascular profile [4,8,10,13]. On the other hand, obesity, the practice of dieting for weight control, inadequate physical activity, and sedentary lifestyle were shown to be related to irregular breakfast eating at this stage of life [2,3,9,10,19–23]. Though, it is important to highlight that causal relationship is not well stablished and most studies have cross-sectional design, portraying these findings still as an open question. Also, few studies evaluated changes in breakfast habits during adolescence and there is no such data in Brazil.

Frequent breakfast eating may also improve diet quality, and maintenance of this behavior in adolescence is consistently associated with higher intake of fiber, calcium, potassium, zinc, iron and several vitamins [2,9,11,13,19], higher consumption of whole grains, fruits, vegetables and milk [4,24], higher adherence to Mediterranean diet [13], and lower consumption of sugar-sweetened beverages, sweets and chips [2,4].

Several factors have been related to breakfast eating. Girls [2,5–7,25] and adolescents from other non-white skin color [11] skip breakfast more often. Socioeconomic factors have been related to breakfast eating, such as type of school [25], parental education [16] material affluence [26,27] and men’s household income [28]. Also, evidence suggest breakfast frequency related to adiposity. Most longitudinal studies have focused on its influence on weight change [8,10,12,16,22], whereas the opposite direction is poorly studied. Despite cross-sectional findings showing obese students more likely to skip breakfast than overweight and non-overweight youth [22,29], as far as we know, our study is the first to evaluate weight status relationship with breakfast eating changes during adolescence.

Some studies showed a reduced frequency of breakfast eating as adolescents get older [5,8,11]. Considering all possible benefits related to breakfast and the lack of information on breakfast changes over time, it is fundamental to observe how this behavior develops during adolescence and factors associated to inform future public health programs and nutrition interventions for this age. Thus, the current study aimed to evaluate changes of breakfast frequency and foods/food groups consumed at breakfast during adolescence, according to factors usually associated with breakfast eating, such as weight status family breakfast and dieting.

## Materials and methods

### Study design and study population

Data of this prospective study were derived from the Adolescent Nutritional Assessment Longitudinal Study (ELANA), a cohort with adolescents from four private and two public schools located in Rio de Janeiro metropolitan area, Brazil. In Brazil, type of school attending students is considered a proxy of socioeconomic status [30–32].

Sixth-graders from 26 classes attending morning or afternoon schools were invited to participate in the study. ELANA was designed to assess factors related to anthropometric trajectory and was approved by the Research Ethics Committee of Institute of Social Medicine of The State University of Rio de Janeiro (certificate number 0020.0.259.000–09). Participation of the students occurred after their legal guardian provided written informed consent for data publication of study participants.

Adolescents with physical or mental disabilities and who were pregnant or lactating were considered ineligible and not included in the study. Of 945 eligible middle school students, 36 refused to participate, 41 were not authorized by their parents and 59 were not found at school on the days of data collection, leading to a study population of 809 adolescents in 2010 (baseline). After three years, 39.6% of the students were lost to follow-up. Reasons for losses to follow-up included missing school on the days of data collection (n = 370), students refusals to participate (n = 99), non-authorization by parents of continuity in the research (n = 55) and pregnancies (n = 3). Thus, the final sample consisted of 488 adolescents in 2013.

### Data collection and measurements

At all stages of the study, data collection of anthropometric data and a self-administered questionnaire were conducted by a previously trained team of research assistants and supervised by post-graduate students, who were responsible for clarifying questions with participants and reviewed all the questionnaires. Data collected were typed by two students and compared to rectify potential typing errors. Respondent’s errors or lack of information were returned to the field for data correction with each participant.

Sociodemographic, weight-related and behavioral data were obtained by application of a self-report questionnaire in each year of data collection. The self-reported questionnaire can be found available at http://www.nebin.com.br/material.html. Breakfast frequency was estimated at baseline and after three years. The respondents were asked how often they ate breakfast and five response options were given: never or almost never, 1 to 2 times a week, 3 to 4 times a week, 5 to 6 times a week, and daily. Response options were collapsed for analyses as less than 5 times a week, for irregular breakfast frequency, and 5 times a week to daily, for regular breakfast frequency. To analyze breakfast skipping, skippers were categorized as those who responded never or almost never, and non-skippers, those with breakfast frequency of 1 or more times a week. Similar categorizations for skipping breakfast were conducted in previous studies [12,33]. The same question and response options were provided for breakfast frequency with parents, categorized in regular and irregular family breakfast frequency.

At baseline and after three years of follow-up a qualitative food frequency questionnaire (FFQ) was applied with the students. The ELANA FFQ was adapted from a FFQ validated for adolescents from Rio de Janeiro metropolitan area, Brazil [34], though no differences were found in correlation coefficients of nutrient consumption between three-day food records and ELANA FFQ and the original FFQ. Pearson correlation coefficients for ELANA FFQ ranged from 0.41 to 0.23 for energy and nutrients adjusted for energy intake (carbohydrates, lipids, protein, calcium and iron), whilst the original FFQ ranged from 0.41 to 0.34.

The ELANA FFQ asks the frequency of consumption in the last three months of 72 foods and beverages. Eight response options for each item are available: less than once a month or never, 1 to 3 times a month, once a week, 2 to 4 times a week, 5 to 6 times a week, once a day, 2 to 3 times a day, 4 or more times a day. For our analyses, the response options were transformed into daily frequency and weights were given for each option, taking into account the average frequency of the response option and time, in days. According to the pattern of distribution of the food groups usually consumed at breakfast, response options were divided in three categories: monthly to no consumption, weekly and daily.

We assessed foods/food groups usually consumed at breakfast according to findings from Correa et al. (2016), who identified dietary patterns regarding breakfast from one 24-hour dietary recall (24hR) with adolescents aged 10 to 14 years old, from public schools of Rio de Janeiro metropolitan area, Brazil [35]. Thus, fourteen foods/food groups were designated from our FFQ: cheese (white cheese, yellow cheese, mozzarella cheese and others), milk, chocolate powders, fruits (banana, pineapple, guava, strawberry, orange, apple and papaya), bread, coffee, ham, chips, cookies, eggs, sweets (candies, chocolate, condensed milk, jelly, jam, peanut candy, ice cream), Sugar sweetened beverages (fruit drinks, guarana refreshment, sodas and tea), and snack and sandwiches (hamburger, hot dog, pizza, deep-fried and baked snacks).

Physical activity energy expenditure (PAEE) was estimated by the short form of the International Physical Activity Questionnaire (IPAQ), validated with Brazilian adolescents of at least 12 years old [36]. Since part of our study sample was younger at baseline, IPAQ was meticulously explained to students and response options were revised. Duration (time per day) and frequency (days per week) of walking, moderate-intensity activities and vigorous-intensity activities were obtained by the eight open-ended questions of IPAQ. Respective time spent per week with walking, moderate and vigorous activities were multiplied by metabolic equivalent of task (MET) intensities, and intensity-time products were summed to generate PAEE [37,38].

We asked the frequency of dieting for weight control in the last three months and response options were never, rarely, often and always. For analyses, we collapsed the options rarely, often and always as practice of dieting and never to represent no dieting. Skin color was obtained according to Brazilian Institute of Geography and Statistics classification [39], and students were divided in white and other skin color for analyses.

Along with the self-report questionnaire, anthropometrics were measured by a previously trained research team, following Lohman et al. (1988) protocols [40], and were standardized according to Habicht (1974) at baseline [41], and Norton and Olds for the follow-up occasions [42]. Adolescents’ weight and height allowed for the calculation of body mass index (BMI). Weight was measured with a portable electronic platform scale (Kratos-cas, Brazil) to the nearest 0.05 kg, and height was measured twice with a stadiometer to the nearest 0.1 cm (Alturexata, Brazil). Mean height was estimated for analyses, and when height variation exceeded 0.5 cm, the measurement procedure was repeated.

Adolescents’ weight status was assessed according to the age and sex-specific cut-off points for BMI, as published by the International Obesity Task Force [43]. We collapsed adolescents classified as adequate and thinness with a BMI-for-age z-score ≤ 1, who became the non-overweight/obese category, and those who had BMI-for-age z-score > 1 corresponded to the overweight/obese category. Thin adolescents joined those classified with adequate BMI-for-age z-score, considering that they were few (n = 22) and primary analyses showed the same direction of response between groups classified as thinness and those adequate. To calculate BMI-for-age z-scores we used WHO-Anthro Plus, version 3.2.2.

### Statistical analyses

All statistical analyses were stratified by sex and were performed using Statistical Analysis System, version 9.4 (SAS Institute Inc., Cary, NC, USA). Cross-sectional analyses between breakfast frequency and sociodemographic, weight-related, and behavioral factors at baseline were estimated using Student t tests to compare means, and chi-square tests to compare proportions. To observe differences between adolescents that participated at baseline and those participating in both baseline and after three years we conducted chi-squared tests for baseline variables. Descriptive data were expressed in percentage, means and standard deviation values.

To address whether breakfast frequency changed after three years and the relationship with baseline characteristics, longitudinal analyses were applied using generalized estimating equations (GEE) with log-binomial models for repeated measures, with estimation of relative risks and 95% confidence intervals (PROC GENMOD from SAS). All analyses included the school cluster effect. The characteristics selected for longitudinal analyses were those statistically significant in cross-sectional analyses. Initially, each model included time, the baseline exposure variable and an interaction term time-exposure, which allowed the evaluation of potential differences between exposure groups in response change over time.

Models were also tested for effect modification by weight status, family breakfast and dieting. Since the interaction term was statistically significant only for weight status, an analysis stratified for weight status is shown. As models with the other exposure variables were not statistically significant for the interaction term, we withdraw the interaction term and conducted a multivariate model including the variable time and adjusted for the exposure variables that were significantly related to the outcomes at baseline. These analyses were conducted for regular/irregular breakfast and repeated for skipping breakfast (skippers vs. non-skippers).

To address changes in foods/food groups usually consumed at breakfast, we selected only the participants who reported breakfast for both time occasions, leading to a study sample of 380 adolescents. We also excluded 11 individuals at baseline with FFQ response inconsistencies. To verify how these foods/food groups changed in three years, time was modeled using generalized estimating equations with multinomial models, with estimation of relative risks and 95% confidence intervals (PROC GENMOD from SAS). All analyses included the school cluster effect.

The advantage of using generalized estimating equations is that analyses account for losses of follow-up for the outcome. This approach can handle time-unbalanced data as a function of missing values for the outcome and no data on individuals at any occasion is discarded [44,45].

## Results

At baseline, approximately a quarter of adolescents consumed breakfast irregularly. Breakfast was skipped by 9.6% of all adolescents. Half of study participants were having irregular breakfast with their parents and a high proportion of them were overweight or obese (42.5%). Almost half of students have been on a diet in a reference period of three months and 37.1% were from public schools. Adolescents were 11.8 years old, on average, aged 10–16 years old at baseline, and age ranged from 12.9 to 18.6 after three years There were no statistically significant differences between adolescents participating at baseline and those who continued participating of the study after three years for breakfast frequency, breakfast skipping, sex, skin color, weight status, family breakfast frequency and dieting. However, mean age and proportion of adolescents from public schools who participated of the study after three years of follow-up were lower than of those who participated at baseline (Table 1).

**Table 1:** Baseline characteristics of adolescents that participated at baseline and both at baseline and after three years of follow-up (3y fu).

Variables	Baseline		3y fu		P-value^a^
	n	%	n	%	
**Sex**	809		488		0.808
Male	435	53.77	259	53.07	
Female	374	46.23	229	46.93	
**Type of school**	809		488		**0.013**
Private	509	62.92	340	69.67	
Public	300	37.08	148	30.33	
**Skin color**	803		484		0.396
White	367	45.70	233	48.14	
Other skin color	436	54.30	251	51.86	
**Weight status**	791		478		0.168
Non-overweight/obese	455	57.52	256	53.56	
Overweight/obese	336	42.48	222	46.44	
**Breakfast frequency**	805		485		0.650
Regular	595	73.91	364	75.05	
Irregular	210	26.09	121	24.95	
**Skipping breakfast**	805		487		0.962
No	728	90.43	422	86.65	
Yes	77	9.57	65	13.35	
**Family breakfast**	803		484		0.377
Regular	402	50.06	230	47.52	
Irregular	401	49.94	254	52.48	
**Dieting**	797		482		0.593
No	424	53.20	249	51.66	
Yes	373	46.80	233	48.34	
	**n**	**mean (sd)**	**n**	**mean (sd)**	**P-value**^b^
**PAEE (MET-minutes/week)**	796	2907 (2750)	478	2778 (2629)	0.409
**Age (years)**	809	11.83 (1.15)	488	11.53 (0.78)	**<.0001**

PAEE, physical activity energy expenditure in MET-minutes/week.

^a^ P-value of chi-squared test

^b^ P-value of Student t test

Breakfast was consumed irregularly among 21.2% (n = 92) of boys and 31.8% (n = 118) of girls. As for skipping breakfast, 7.8% (n = 34) of boys and 11.6% (n = 43) of girls were skippers. Differences at baseline in irregular breakfast frequency and breakfast skipping according to socioeconomic, weight-related and behavioral characteristics, stratified by sex, are shown in Table 2. Both boys and girls eating breakfast irregularly and skipping breakfast were more likely to have irregular family breakfast. At baseline, no statistically significant differences of irregular breakfast eating and of breakfast skipping were found for type of school, skin color, physical activity energy expenditure and mean age for both boys and girls. Girls having irregular breakfast were more likely of being overweight/obese and practicing dieting than girls consuming breakfast regularly.

**Table 2:** Total sample size, frequency (%; n) and mean (sd) of irregular breakfast eating and skipping breakfast at baseline according to socioeconomic, weight-related and behavioral aspects of adolescents, by sex.

Variables^a^	Boys			Girls		
	Total	Irregular Breakfast	Breakfast Skipping	Total	Irregular Breakfast	Breakfast Skipping
		% (n)	% (n)		% (n)	% (n)
**Age group (years)**						
10 to 11	290	20.34 (59)	8.28 (24)	284	30.99 (88)	10.21 (29)
12 or more	144	22.92 (33)	6.94 (10)	87	34.48 (30)	16.09 (14)
**Type of School**						
Private	161	22.34 (61)	8.06 (22)	139	33.62 (79)	11.49 (27)
Public	274	19.25 (31)	7.45 (12)	235	28.68 (39)	11.76 (16)
**Skin color**						
White	203	18.23 (37)	6.90 (14)	164	30.67 (50)	10.43 (17)
Other skin color	228	23.68 (54)	8.77 (20)	208	32.69 (68)	12.50 (26)
**Weight status**						
Non-overweight/obese	235	19.57 (46)	7.66 (18)	220	26.27 (57)	10.60 (23)
Overweight/obese	192	23.44 (45)	8.33 (16)	144	40.28 (58)**	13.89 (20)
**Family breakfast**						
Regular	237	9.28 (22)	3.80 (9)	165	12.73 (21)	5.45 (9)
Irregular	196	35.71 (70)***	12.75 (25)**	205	46.83 (96)***	16.59 (34)**
**Dieting**						
No	226	21.68 (49)	8.41 (19)	198	24.24 (48)	8.08 (16)
Yes	205	20.98 (43)	7.32 (15)	168	39.29 (66)**	14.29 (24)
**PAEE**						
1^st^ tercile	119	19.33 (23)	6.72 (8)	148	34.46 (51)	12.84 (19)
2^nd^ tercile	141	22.70 (32)	9.22 (13)	126	30.16 (38)	11.11 (14)
3^rd^ tercile	174	21.26 (37)	7.47 (13)	97	29.90 (29)	10.31 (10)

PAEE, physical activity energy expenditure in MET-minutes/week divided in terciles; Irregular breakfast, breakfast < 5 times/week; Skipping breakfast, breakfast as never or almost never.

^a^ Variables at baseline.

** P-value<0.001;

*** P-value<0.0001

Table 3 shows change in irregular breakfast eating over time. Girls had about 30% higher risk of having irregular breakfast after three years (RR = 1.29, 95% CI: 1.08, 1.54). There was no significant effect of interaction between time vs. family breakfast frequency and time vs. dieting, meaning that changes over time were not influenced by family behavior or dieting at baseline. Thus, interaction terms were withdrawn from dieting and family breakfast since the smallest p-values for both irregular breakfast and skipping were 0.11. There was, however, an interaction effect between time and weight status for irregular breakfast. As there was a significant difference for girls that did not appear for boys in the interaction term for irregular breakfast (and not for skipping), we stratified the irregular breakfast for weight status.

**Table 3:** Relative risks (RR) of irregular breakfast after three years of follow-up.

Variables	Boys		Girls	
	RR	95% CI	RR	95% CI
**Multivariate model** ^a^				
Time	1.13	0.89, 1.43	1.29	1.08, 1.54
Dieting	0.97	0.73, 1.28	1.27	1.01, 1.60
Irregular family breakfast	3.13	2.24, 4.37	2.30	1.77, 3.01
**Models stratified by BMI classification** ^b^				
Non-overweight/obese	0.72	0.47, 1.11	1.54	1.17, 2.04
Overweight/obese	1.40	1.03, 1.89	1.11	0.88, 1.39

^a^ No effect for time*exposure interaction for the variables included in the model

^b^ Models adjusted for dieting and family breakfast frequency

Among those who were overweight/obese at baseline, breakfast change was influenced differentially according to sex. Thus, boys overweight/obese at baseline had a 40% increase of irregular breakfast, whereas among girls this increase occurred only among those not overweight/obese at baseline (RR = 1.54, 95% CI: 1.17, 2.04). When skipping breakfast was analyzed (Table 4), there was no significant effect of interaction between time and any of exposure variables. Girls had 63% higher risk of skipping breakfast after three years (RR = 1.63, 95% CI: 1.12, 2.38), without increase among boys.

**Table 4:** Relative risks (RR) of skipping breakfast change after three years of follow-up.

Variables	Boys		Girls	
	RR	95% CI	RR	95% CI
**Multivariate model** ^a^				
Time	1.11	0.68, 1.82	1.63	1.12, 2.38
Dieting	0.79	0.46, 1.38	1.39	0.83, 2.35
Irregular family breakfast	2.38	1.36, 4.14	1.92	1.20, 3.09
Weight status	1.29	0.73, 2.30	0.88	0.53, 1.45

^a^ No effect for time*exposure interaction for the variables included in the model.

Cross-sectional analyses of frequencies of foods/food groups usually consumed at breakfast indicated that the consumption of most items were reduced after three years of follow-up, except for sugar-sweetened beverages, eggs and sweets, that had similar consumption after three years, and eggs, that increased (Table 5). Longitudinal analyses of foods/food groups change over time revealed that boys had higher risk of reducing consumption of fruits, chips, sandwiches and snacks, and ham after three years, lower of consumption of cheese, and change was not statistically different for milk, bread, eggs, cereals, cookies, sweets, sugar-sweetened beverages, coffee and chocolate powder (Table 6). Girls had higher risk of reducing consumption of fruits, milk, chocolate powder and ham after three years, while there were reductions of consumption for bread, chips, cereals, cookies, coffee and cheese, but estimates were not statistically significant. Risk of increased consumption after three years was observed, though not significant, of sandwiches and snacks, sugar-sweetened beverages and sweets (Table 6).

**Table 5:** Frequencies of foods/food groups usually consumed at breakfast at baseline and after three years of follow-up (3y fu) (n = 380).

Foods/food groups	Baseline		3y fu		P-value^a^
	n	%	n	%	
**Fruits**					<.0001
Monthly to no consumption	41	10.79	51	13.42	
Weekly	101	26.58	152	40.00	
Daily	238	62.63	177	46.58	
**Milk**					<.0001
Monthly to no consumption	80	21.05	90	23.68	
Weekly	88	23.16	117	30.79	
Daily	212	55.79	173	45.53	
**Bread**					<.0001
Monthly to no consumption	45	11.84	35	9.21	
Weekly	129	33.95	159	41.84	
Daily	206	54.21	186	48.95	
**Cereals**					0.002
Monthly to no consumption	234	61.58	248	65.26	
Weekly	85	22.37	94	24.74	
Daily	61	16.05	38	10.00	
**Cookies**					<.0001
Monthly to no consumption	145	38.16	158	41.58	
Weekly	136	35.79	144	37.89	
Daily	99	26.05	78	20.53	
**Chips**					<.0001
Monthly to no consumption	188	49.47	212	55.79	
Weekly	131	34.47	131	34.47	
Daily	61	16.05	37	9.74	
**Sandwiches and snacks**					<.001
Monthly to no consumption	39	10.26	41	10.79	
Weekly	226	59.47	244	64.21	
Daily	115	30.26	95	25.00	
**Sweets**					<.0001
Monthly to no consumption	24	6.34	17	4.47	
Weekly	137	36.05	140	36.84	
Daily	219	57.63	223	58.68	
**Coffee**					<.0001
Monthly to no consumption	233	61.32	234	61.58	
Weekly	60	15.79	86	22.63	
Daily	87	22.89	60	15.79	
**Chocolate powder**					<.0001
Monthly to no consumption	100	26.32	106	27.89	
Weekly	103	27.11	133	35.00	
Daily	177	46.58	141	37.11	
**Sugar-sweetened beverages**					0.005
Monthly to no consumption	7	1.84	10	2.63	
Weekly	85	22.37	78	20.53	
Daily	288	75.79	292	76.84	
**Cheese**					<.001
Monthly to no consumption	141	37.11	106	27.89	
Weekly	136	35.79	186	48.95	
Daily	103	27.11	88	23.16	
**Ham**					<.0001
Monthly to no consumption	128	33.68	155	40.79	
Weekly	144	37.89	163	42.89	
Daily	108	28.42	62	16.32	
**Eggs**					<.001
Monthly to no consumption	192	50.53	187	49.21	
Weekly	172	45.26	153	40.26	
Daily	16	4.21	40	10.53	

^a^ P-value of chi-squared test

**Table 6:** Relative risks (RR) of reducing intake of foods usually consumed at breakfast from baseline to three years of follow-up.

Foods/food groups	Boys		Girls	
	RR	95% CI	RR	95% CI
**Fruits**	1.60	1.20, 2.13	2.08	1.47, 2.95
**Milk**	1.33	0.97, 1.83	1.49	1.07, 2.08
**Bread**	0.95	0.69, 1.32	1.40	0.96, 2.04
**Cereals**	1.26	0.89, 1.79	1.23	0.83, 1.82
**Cookies**	1.25	0.92, 1.70	1.20	0.89, 1.63
**Chips**	1.43	1.01, 2.03	1.32	0.96, 1.83
**Sandwiches and snacks**	1.58	1.12, 2.22	0.89	0.61, 1.31
**Sweets**	1.07	0.78, 1.47	0.73	0.50, 1.07
**Coffee**	1.08	0.82, 1.44	1.19	0.85, 1.68
**Chocolate powder**	1.15	0.84, 1.57	1.54	1.11, 2.14
**Sugar-sweetened beverages**	0.99	0.67, 1.48	0.90	0.56, 1.44
**Cheese**	0.73	0.53, 1.00	1.07	0.75, 1.52
**Ham**	1.52	1.12, 2.07	1.65	1.16, 2.36
**Eggs**	1.02	0.72, 1.44	1.42	1.00, 2.01

## Discussion

Our findings revealed that girls who skipped breakfast or had breakfast irregularly at the beginning of the study had higher risk of continue with these patterns after three years. Among boys, having breakfast irregularly or skipping this meal at baseline did not seem to be related to breakfast eating by the end of the study. Studies using data from the National Longitudinal Study of Adolescent Health found that Americans eating breakfast regularly at adolescence were more likely to maintain this pattern during young adulthood, in Merten et al. (2009) study [15], and breakfast eating decreased from adolescence to early adulthood, in Niemeier et al. (2006) study [46]. Although the analyses from Merten et al. (2009) were controlled for sex, no stratified analyses or modification effect were investigated [15]. Pedersen et al. (2013) reported that having low breakfast frequency (categorized as more seldom than daily) at 15 years predicted low breakfast frequency at 19 years that, in turn, predicted low breakfast frequency at 27 years [47]. Further, low breakfast frequency at 15 years predicted low breakfast frequency at 27 years. Same patterns were found in analyses stratified by sex, but the observed predictions between 15 and 27 years for men were not significant. To some extent, their findings are consistent with our observations.

Weight status was related to change in breakfast consumption over time. Overweight/obese boys and non-overweight/obese girls having breakfast irregularly at baseline had greater risk of having irregular breakfast after three years. As far as we know, no research of weight status modification effect on breakfast eating change over time was conducted. Though, longitudinal studies of breakfast eating influence on adiposity and weight status are better documented, despite no consistent evidence [22,33,48]. Berkey et al. (2003) investigated the modification effect of weight status and sex on the relation of breakfast history and BMI change among adolescents aged 9–14 years at baseline [33]. They found overweight children and breakfast skippers over the previous year with smaller BMI increase, relative to peers who were daily breakfast eaters. Though it was not significant, normal weight children gained weight relative to eaters. The National Heart, Lung, and Blood Institute Growth and Health Study (NGHS) observed that only among American girls with a high BMI-for-age at baseline, frequent breakfast eating was associated with decreased BMI after 10 years [49]. Other studies should further explore the combination of previous weight status, weight change and change in breakfast eating to clarify these associations.

When the outcome of interest was skipping breakfast, weight status did not appear as a modifying effect. Among girls, the risk of skipping breakfast in three years was of higher magnitude than the risk of having irregular breakfast in the same period. Dialektakou and Vranas (2008) compared 24 different definitions and categorizations of breakfast skipping among Greek high school students [50]. The authors observed that results changed depending on how breakfast skipping was defined and categorized. For example, when adolescents were asked if they had eaten any solid food in the morning of the interview, 39% responded negatively. By adding the consumption of any beverage to the question the prevalence of skipping breakfast fell by 12.5%. When argued if they did not eat breakfast weekly throughout the year, 8.4% were considered skippers, whilst 3.6% skipped the meal when the reference period was the last week. Additionally, the authors found some breakfast skipping definitions associated with BMI and weight status, but others were not. Given this, the findings can vary widely, which may compromise the comparison between studies.

It is evident that how one defines and categorizes breakfast can significantly influence findings and their interpretation. For this reason, further discussion on this matter and future research attempting to establish a standardized definition of what constitutes a breakfast in scientific literature is essential. This may allow better comparisons across studies and interpretation of the existing literature, and provide appropriate methods to advance the field that could lead to better grounded recommendations for population.

Among breakfast eaters we found higher risk of decreased consumption of fruits and ham for both sexes; chocolate powder and milk for girls; chips, sandwiches and snacks for boys. Cheese was the only food group with higher probability of lower consumption after three years, but only among boys. Also, we found no statistically significant changes in three years for consumption of bread, coffee, cereals, cookies, sugar-sweetened beverages and sweets for boys and girls; cheese, chips, and sandwiches and snacks for girls; milk, eggs and chocolate powder for boys. Few studies have tried to characterize breakfast over time. Lytle et al. (2000) followed eating patterns in students from Minnesota at the third, fifth, and eighth grades [1]. Their findings showed that the pattern of breakfast across three-time periods shifted to a less healthful dietary behavior, with declined variety of foods in this meal. Daily fruit, vegetable and salty snacks consumption fell by the end of the study. The proportion of beverage from milk and fruit juice consumption also had fallen, whilst sodas contribution nearly tripled.

It is important to highlight that we captured foods usually consumed at breakfast from Correa et al. (2016), who captured breakfast patterns from 24hR. As our data was collected from a FFQ, we had limited food options compared to those given by a 24hR and, thus, adaptions were necessary [35]. Besides, the FFQ captures daily food consumption, and foods/food groups chosen in the current study may be consumed in eating occasions other than breakfast. Therefore, these foods/food groups may not reflect foods actually consumed at breakfast, which reflects one limitation of the current study.

Despite this limitation, there is certain homogeneity between urban regions of western countries with regards to breakfast patterns, usually composed by some type of milk, bread and cereals [17]. Similar breakfast patterns were observed in studies with adults from São Paulo, Brazil [51,52]. Santos et al. (2015) revealed three eating patterns at breakfast: traditional, linked to coffee, sugar, butter/margarine and bread/toast; healthy, characterized by fruits, whole-grain bread, skimmed milk/semi skimmed milk and white cheese; and a snack pattern, correlated with consumption of cold cuts, bread/toast and yellow cheese in this meal [51]. Similar to these findings, Mattos and Martins (2000) showed breakfast composed mainly of coffee, bread, sugar, margarine/butter, whole milk, and also, fruits and cheese [52]. Despite these similarities, breakfast patterns of Brazilian adolescents are not well documented and should be further explored.

Studying adolescents from Rio de Janeiro metropolitan region, Brazil, Correa et al. (2016) reported that most consumed foods/food groups at breakfast were: breads (64%), sugar and sweets (58%), butter or margarine (50%), milk and milk-based beverages (49%), and coffee and tea (34%) [30], similar to breakfast patterns found in Brazilian adult’s studies [51,52]. In contrast to those studies, chocolate milk appeared as most consumed by adolescents (37%), while cheese (12%) and fruit (4%) were less consumed. Also, beverages marked differences in breakfast patterns, mainly by three patterns: sugar-sweetened beverages, milk and milk-based beverages consumption, and coffee and tea.

It is necessary to further understand the composition of breakfast to improve diet quality among adolescents, since they present unhealthy eating habits and higher nutritional requirements, as a result of intense physical growth at this stage of life [25,53,54]. Data from Brazilian studies revealed that approximately 20% of ninth grade middle school students did not consume milk, fruits and vegetables a week prior to data collection [25] and, compared to adults, Brazilian adolescents presented higher consumption of sodas, biscuits and sausages, and lower of vegetables and beans [55,56]. Another nationally representative study from Brazil showed that almost all adolescents had intake of calcium below the recommendation for the age group [54]. As breakfast eating is related to higher intake of several nutrients, such as calcium and vitamin D [9,11,19] and, thus, higher growth potential can be achieved, investments in policies that improve breakfast eating are needed.

One possible explanation for the changes we observed for breakfast frequency and composition may be due to the fact that adolescents get more autonomy and responsibilities as they grow, which includes changing lifestyle and dietary habits [57,58]. Along with that, parent’s participation in their breakfast also decreases as they grow older [59]. There is a growing body of evidence showing frequent family meal to be related with regular breakfast eating and overall improved diet quality [20,59,60]. We found a significant association between these behaviors in our cross-sectional analyses, and irregular family breakfast at baseline was related to increased risk of both skipping and irregular breakfast frequency. However, there was no effect of interaction between time and family breakfast frequency.

It is important to highlight that significant differences in type of school proportions between participants present at the baseline and those present in the two time points of our study can be explained by the fact that student’s evasion from schools was greater among adolescents from the public ones, as they started to work or entered military service. Also, students from public schools are older and are the ones who most left of our study, which explains the significant differences between baseline age averages of students participating at baseline and those who continued participating after three years. Therefore, differences between the losses according to type of school and age may not be related to breakfast eating pattern, which speaks against the possibility of a pattern of missing related to the outcome. Also, despite the high percentage of ELANA losses, all longitudinal modeling accounts for losses for the outcome, and the exposure variables were analyzed at baseline.

This study is limited in what concerns external validity, and thus, other longitudinal cohort with larger and more representative samples of adolescents from Brazil can corroborate to further explore this scenario. Also, more research is necessary with regards to explore how breakfast pattern change over time. Our findings showed that in three years student`s breakfast composition tend to decrease for some foods/food groups, while maintain a certain stability for others.

Our study represents advancement in the knowledge of breakfast frequency and composition and factors related to its consumption, especially the role of weight status on breakfast change. Given our findings of higher risk of breakfast to be less consumed among girls and that breakfast composition worsened as adolescents grow old, it is fundamental to encourage its consumption in this age group, once breakfast eating is recognized to be related to healthier food consumption and several other health aspects [6,9–11,13,18,19]. The promotion of heath programs considering the school environment could help changing this scenario. In Brazil, adolescents attending public schools have been receiving lunch as part of the Brazilian School Feeding Program, a successful public policy implemented in 1954 [61]. Objectively, considering breakfast inclusion in this program or, if not possible, planning intervention strategies through health education activities that stimulate breakfast eating could promote healthier food consumption among this age group.

In the public health perspective, our findings can also contribute to subsidize future dietary guidance for Brazilians, since the last Food Guideline for Brazilian Population, published in 2014, valorized eating and composition of the three main meals: breakfast, lunch and dinner. In Brazil, there is still lack of information on adolescent`s breakfast patterns and our findings contribute to fulfill this gap.

## Supporting information

S1 File Data set for foods/food groups analyses. (XLSX)

S2 File Data set for breakfast frequency analyses. (XLSX)
